# The clinical consequences of sucrase-isomaltase deficiency

**DOI:** 10.1186/s40348-015-0028-0

**Published:** 2016-02-08

**Authors:** Stanley A. Cohen

**Affiliations:** Children’s Center for Digestive Health Care, Children’s Healthcare of Atlanta, 993D Johnson Ferry Road NE, Suite 440, Atlanta, GA 30342 USA

**Keywords:** Sucrase, Sucrase-isomaltase deficiency, Sacrosidase, Genetic sucrase-isomaltase deficiency, Heterozygous carriers

## Abstract

Primary sucrase-isomaltase deficiency, originally thought to be a homozygous recessive disorder, has been found to have numerous genetic variants that alone or in combination (compound heterozygosity) express varying degrees of clinical illness, most commonly causing chronic diarrhea, abdominal pain, and bloating. These symptoms are also present with secondary sucrase-isomaltase deficiency. Recent investigations are providing evidence that sucrase-isomaltase deficiency is more prevalent and of greater clinical significance than previously suspected. Further research is required to correlate the specific genotypes and phenotypes with their clinical expressions and to determine the most appropriate treatment algorithm for these patients.

## Introduction

The linked disaccharidase, sucrase-isomaltase, is a glycoprotein localized to the brush border membrane of small intestinal villi. Sucrase-isomaltase is synthesized and assembled in the rough endoplasmic reticulum as a homologous pro-enzyme dimer which passes through the Golgi apparatus and is transported to the apical cell surface of villi [1]. There, it is cleaved into its mature subunits, sucrase and isomaltase, by pancreatic proteases. Isomaltase cleaves branched (1–6 linked) α-limit dextrins, while sucrase digests sucrose, maltose, short 1–4 linked glucose oligomers, and some branched starches into sugar monomers for intestinal absorption, its activity often overlapping with maltase-glucoamylase [2]. The cleaved monosaccharides are then transported across the epithelial brush border for absorption and metabolism. Sucrase is an inducible enzyme in early development, such that increased exposure to sucrose, or the stress of doing so, increases sucrase activity [3–7].

## Review

### Deficiency states

The first report of an autosomal recessive disaccharidase deficiency was congenital sucrase-isomaltase deficiency (CSID) in 1960 [8]. The affected children presented with osmotic diarrhea, mild steatorrhea, chronic diarrhea, irritability, and vomiting after consuming sucrose [9]. The difficulty in providing adequate nutrition to these patients can lead to dehydration, metabolic acidosis, hypercalcemia, failure to thrive, and developmental delay. CSID was originally believed to be rare, occurring primarily among the native population of Alaska and Greenland [10, 11]. A trafficking defect with intracellular accumulation of pro-sucrase-isomaltase in the Golgi apparatus was found to be responsible [1].

## Genetic factors

Since the discovery of the initial trafficking error, other phenotypes have been discovered [12] and, currently, seven phenotypes are known [13]. More than 25 mutations within the human sucrase gene are responsible for these CSID phenotypes [14]. Some sucrase-isomaltase variants show classical autosomal recessive homozygous inheritance, while others demonstrate compound heterozygote inheritance [15]. Sucrase-isomaltase variants can occur on either sucrase or isomaltase subunits, resulting in varied effects on sucrase-isomaltase enzyme activity [16].

There is now strong evidence that heterozygous carriers also experience symptoms of CSID, such as chronic diarrhea, abdominal pain, and bloating [17]. It is currently estimated that 2–9 % of Americans of European descent may be affected, suggesting that sucrase-isomaltase deficiency has been greatly underrecognized [17]. Therefore, the newer term genetic sucrase-isomaltase deficiency (GSID) should be used to encompass the entire range of inheritance patterns while CSID is reserved to specifically identify homozygous recessive individuals.

For example, the sucrase-isomaltase variants V577G and G1073D result in blocked transport in the ER loss of normal enzyme activity while the sucrase-isomaltase variant V15F has reduced cell surface expression resulting in decreased sucrase activity [16]. Analyses at the cellular, molecular, and functional levels of 11 novel cases with CSID revealed three categories of sucrase-isomaltase variants: transport competent, partially transport competent, and transport incompetent [18]. Patients with all three variant categories showed reduced or drastically reduced enzymatic activities of sucrase and isomaltase and symptoms resembling irritable bowel syndrome [17].

## Secondary or acquired sucrase-isomaltase deficiency

Available evidence suggests that other forms of chronic diarrhea share some of the features of GSID and may represent acquired or secondary forms of sucrase-isomaltase deficiency. For example, a reduced height of intestinal villi in pigs is directly correlated with reduced brush-border enzyme activity, specifically lactase, sucrase, and maltase [19]. Clinically, the reduced enzymatic activity and villous atrophy in the small intestine are associated with maldigestive and malabsorptive diarrhea [20]. Numerous human gastrointestinal disorders, such as celiac disease, are also associated with villous atrophy and diarrhea and may represent secondary or acquired forms of sucrase-isomaltase deficiency (Table 1) [21]. In these disorders, the clinical impact of sucrase-isomaltase deficiency is often transient, with enzymatic activity gradually returning to normal or near normal as the underlying disorder is successfully resolved.

**Table 1:** Potential causes of secondary or acquired sucrase-isomaltase deficiency or maldigestion

Villous atrophy or alteration
Celiac disease
Non-tropical sprue
Chemotherapy and radiation enteropathy
Crohn’s disease
Allergic enteropathy
Immunodeficiency
Malnutrition
Infection
Acute gastroenteritis
Giardiasis
Tropical sprue
HIV enteropathy
Small intestinal bacterial overgrowth
Rapid transit
Rapid gastric emptying
Chronic nonspecific diarrhea
Dumping syndrome
Ulcerative, microscopic, and lymphocytic colitis

## Clinical features

Whether due to a primary (genetic) or secondary (acquired) deficiency, absent or diminished enzyme activity allows undigested sugars to accumulate in the lumen of the small intestine, resulting in the clinical effects of sucrase-isomaltase deficiency [12]. The osmotic effect of malabsorbed sugars contributes to watery, hyperosmolar diarrhea. Subsequently, other symptoms are caused by the fermentation of undigested sucrose by colonic bacterial microflora which release methane, hydrogen, and carbon dioxide causing bloating and abdominal pain [22].

Symptom severity becomes a function of residual sucrase and isomaltase activity, amount of sugar and starch consumed, extent of buffering by other foods, and gastric emptying [22]. Frequent diarrhea can be the result and also the cause of rapid small-bowel transit. The degree of fermentation of any malabsorbed carbohydrates by the colonic bacteria can also stimulate diarrhea and further lessen exposure time for existing enzymes to digest substrate [22]. Children are more susceptible to the symptoms of sucrase-isomaltase deficiency because the length of their small intestine is shorter and the reserve capacity of the colon to absorb excess luminal fluid is reduced [22]. Consequently, symptoms sometimes improve with age.

The current standard for the diagnosis of sucrase-isomaltase deficiency is to assay duodenal or jejunal mucosa biopsy specimens for lactase, sucrase, isomaltase (palatinase), and maltase activity [22, 23]. In addition to the usual histological specimens obtained at the time of upper endoscopy, several biopsies are sampled from the distal duodenum or jejunum, immediately frozen and sent to a laboratory, and assayed for enzyme activity. The standard assay is the Dahlquist method which incubates the biopsied tissue together with the appropriate substrate and measures the released glucose. Using this method, disaccharidase activity in the duodenum can be almost 40 % less than that in the proximal jejunum [24]. Additionally, the broad effectiveness of SI in digesting 1,4-linkages results in a tight correlation of sucrase activity with maltase and isomaltase activity [25].

The invasiveness of obtaining biopsies for disaccharidase assays has led to the development of surrogate methods, based on the exhalation of gases produced by the enzymatic degradation of the substrate which reflect the clinical effect of sucrose intolerance. The sucrose hydrogen breath test is easily performed by having the patient drink a standard amount of sucrose or ^13^C-sucrose. The amount of expired hydrogen or ^13^C-methane measured in expired breath correlates well with intestinal enzyme activity [26]. More recently, sucrase-isomaltase exome genetic sequencing has become available for identifying homozygous and compound heterozygote mutations responsible for genetic sucrase-isomaltase deficiency [16]. Additionally, dietary modification and oral enzyme replacement trials using synthetic sucrase (sacrosidase) have been employed to determine if they can clinically alleviate symptoms of sucrase-isomaltase deficiency [22, 23].

## Growing prevalence of sucrase-isomaltase deficiency

A retrospective study assessed levels of disaccharidase activity in mucosal biopsy tissue samples referred to a large gastrointestinal specialty laboratory over a 6-year period (*N* = 27,875) [27]. These specimens, most of which were collected during pediatric endoscopies, revealed at least one disaccharidase deficiency in 45 % of samples. Among these, 21 % were sucrase deficient, indicating that 9.3 % of the specimens showed low sucrase activity.

**Fig. 1:**
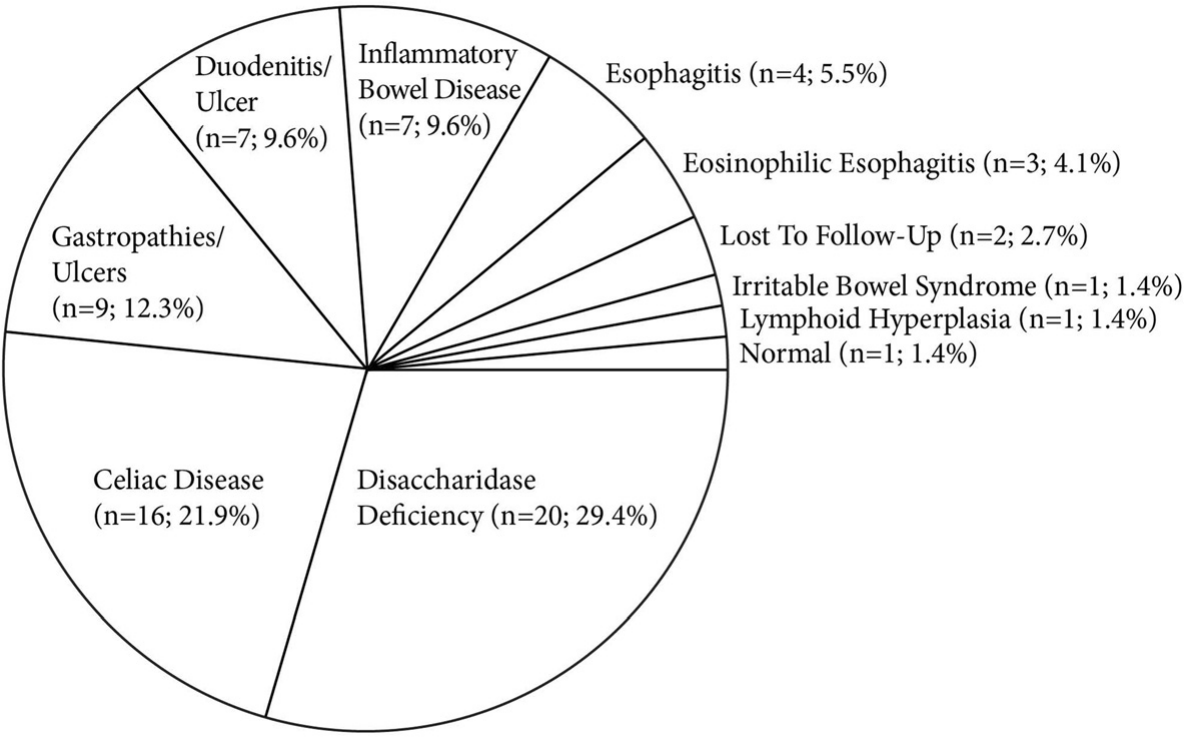
Primary diagnosis of symptomatic children undergoing upper endoscopy and disaccharidase testing. Final diagnosis on the visit after EGD and disaccharidase testing among 73 children who were sucrase deficient (enzyme activity <25 μmol/min/g)

Subsequently, a prospective pilot study assessed the prevalence of disaccharidase abnormalities in children with recurrent abdominal pain undergoing upper endoscopy (esophagogastroduodenoscopy (EGD)) (*N* = 28). Among them, 15 (53.6 %) had low lactase levels, 4 (14.3 %) had low sucrase levels, 5 (17.9 %) had low maltase, and 4 (14.3 %) had low glucoamylase activity. Clinical characteristics did not correlate with the degree of disaccharidase deficiency [28]. More recently, a retrospective review studied 963 symptomatic children undergoing upper endoscopy and disaccharidase testing. Among those, 73 (7.6 %) were sucrase deficient, defined as enzyme activity <25 μmol/min/g. Only 4 children (5 %) had isolated sucrase-isomaltase deficiency, with the others also having lactase deficiency or pan-disaccharidase deficiency when all enzymes were measured (*n* = 44; 60 %). Abnormal biopsies were found in 33 children (45 %). Most had abdominal pain (*n* = 49; 78 %) and/or diarrhea (*n* = 27; 43 %). The final diagnosis for these 73 patients is shown in Fig. 1. It is important to note, however, that even though disaccharidase deficiencies were found on biopsy, the attending physician may not have accepted the deficiency as the primary diagnosis [25].

Thus, there is still concern that the cited studies underestimate the prevalence and only comprise a limited view of SID. In a study of 65 children diagnosed with GSID, 53 reported the onset of symptoms before 1 year of age; however, only 17 were diagnosed by that time and 30 were not diagnosed until 5 years of age [19]. While some are correctly diagnosed, many others may be missed due to dietary elimination of high sucrose or high starch foods as food allergies or misdiagnosed as chronic nonspecific diarrhea or diarrhea-predominant irritable bowel syndrome. As a result, it is incumbent upon us to more fully understand the clinical consequences of SID. Population-based and well-controlled studies are needed to explore the relationship of the various genetic and secondary deficiencies and correlate them with their clinical manifestations and response to therapy.

## Conclusions

Sucrase-isomaltase deficiency is more prevalent than previously suspected and often contributes to significant symptoms for patients with either primary or secondary disease. Further research is required to correlate the genotype with a phenotype and to determine the appropriate treatment algorithm for these patients.
